# Mechanical and Thermal Properties of Functionally Graded Polyolefin Elastomer Foams

**DOI:** 10.3390/polym14194124

**Published:** 2022-10-01

**Authors:** Ehsan Rostami-Tapeh-Esmaeil, Sahar Shojaei, Denis Rodrigue

**Affiliations:** Department of Chemical Engineering, Université Laval, Quebec, QC G1V 0A6, Canada

**Keywords:** polyolefin elastomers, graded foams, morphology, mechanical properties, thermal insulation

## Abstract

In this work, uniform and graded polyolefin elastomer (POE) foams were prepared using a single-step technology based on a fixed chemical blowing agent (azodicarbonamide) concentration of 4 phr (parts per hundred rubber). The effect of molding temperature, including the average temperature (*T_avg_*) and temperature difference (Δ*T*), on the foams’ morphology, mechanical properties (tension, compression and hardness) and thermal conductivity was investigated. Two series of samples were produced by fixing *T_avg_* with different Δ*T* or setting different Δ*T*, leading to different *T_avg_*. The morphological analyses showed that two or three regions inside the foams were produced depending on the molding conditions, each region having different cellular structure in terms of cell size, cell density and cell geometry. The results obtained for the conditions tested showed a range of density (0.55–0.72 g/cm^3^), tensile modulus (0.44–0.70 MPa) and compression elastic modulus (0.35–0.71 MPa), with a thermal conductivity between 0.125 and 0.180 W/m.K. Based on the information provided, it can be concluded that the foam’s properties can be easily controlled by the cellular structure and that graded samples are more interesting than uniform ones, especially for thermal insulation applications, such as packaging, construction, transportation, automotive and aerospace industries.

## 1. Introduction

In today’s world, with fast economic and social development, human needs for more sources of energy have become more apparent [1,2]. This is why several practical solutions to generate and/or save energy have been proposed to address these requirements. Some options are renewable energy to reduce our dependence on petroleum fuels and their derivatives, but most of these strategies failed to meet the expected results and only a small number has actually yielded beneficial outcomes [3]. As a result, the need for low-weight materials and energy insulation has reduced material usage and energy loss, leading to economic and environmental advantages. This is why polymer foams have attracted more attention due to their ability to decrease emissions, conserve energy and save materials [4,5,6]. Jahani et al. developed polycarbonate (PC) foams with up to eight-fold expansion ratios and 85% open-cell content for sound and thermal insulation [7]. The group of Vahidifar produced sound insulators based on natural rubber (NR)/nanoclay (NC)/nanocarbon black (NCB) foams with high reflection/absorption coefficient ratio (90%) [8]. Peng et al. improved the sound absorption efficiency of silicon rubber (SR) foams in the presence of NaCl (pore forming agent) for the middle frequency range (1000–2000 Hz), which was related to enhanced resonance matching caused by the open-cell morphology [9].

Today, polymeric foams are widely used in numerous practical fields, such as packaging, automobile, transportation, aeronautic, building and construction, due to their low density and high-energy damping ability, as well as their low thermal, sound and electrical conduction [10,11,12]. To this end, the Dileep’s group reported that functionally graded high-density polyethylene (HDPE) foams exhibited high-energy absorption compared to neat HDPE foam [13].

Polymeric foams, as one of the most important thermal insulators, can be applied to reduce energy losses [14,15,16]. In insulating foams, the heat is mainly transferred by conduction through the solid matrix and gas phase, as well as thermal radiation over the entire volume [17]. Therefore, the chain mobility of a matrix has a significant effect on its thermal conductivity [18]. Since amorphous domains predominate in thermoplastic elastomer materials [19], the vibrational modes of the chains are limited due to the long molecular length and their high level of entanglements, both decreasing the thermal conductivity. On the other hand, trapping gas molecules inside small foam cells, as the lowest heat conduction materials, limits heat transfer by convection [20]. This is why the foam structure and morphology, such as the cell size and cell size distribution, are important factors to determine the overall (macroscopic) thermal insulation behavior [21]. Recent works also claimed that the spatial distribution and gradient of cell size can have a significant effect on these foam characteristics [22,23].

The generation of a cell size gradient inside a foam can be associated with variation in the foaming agent concentration or temperature across the foam thickness under production [24,25,26,27,28,29,30,31]. This asymmetric spatial feature can lead to superior results in terms of mechanical properties and thermal insulation, which can make them useful in a variety of applications, including thermal or sound insulation, high strength at low weight and impact resistance [24,32,33,34]. For example, Gupta fabricated a functionally graded epoxy resin foam using microballoons with different wall thickness as hollow cells. The results showed that the mechanical properties of the foams, such as compressive modulus, strength and total energy absorption, were directly related to the size of the microballoons [35]. In another work, Zhou et al. prepared a functionally graded polymeric foam by controlling the supercritical CO_2_ (ScCO_2_) concentration profile inside polymethyl methacrylate (PMMA) [36]. Their results showed a striking toughness improvement despite the low flexural modulus. Polylactic acid (PLA) open-cell foams with a gradient of pores (200–600 µm) was shown to exhibit a 20% improvement in acoustic absorption capacity compared with uniform foams having the same amount of porosity [37].

Although several reports on functionally graded polymer foams were published, very few of them discussed the thermal insulation properties, especially for elastomer foams. Therefore, the main objective of this work is to produce density-graded thermoplastic rubber foams through careful control of the processing temperature to minimize the thermal conductivity of these elastomer foams. Moreover, in most of the works on polyolefin elastomer (POE) foams, a curing agent was used to better control the foaming process (nucleation and growth steps), while also improving the final mechanical properties. However, this makes the materials more difficult to recycle. Hence, in this work, the foams were prepared by using poly (ethylene-*co*-octene) as the matrix and azodicarbonamide (ADC) as a chemical foaming agent without adding a curing agent. Then, the graded foams were produced under a temperature gradient using different average temperature (*T_avg_*) and temperature difference (Δ*T*) to compare with uniform foams (constant density across thickness). To complete the analysis, mechanical properties (tension, compression and hardness) are also included. To limit the experimental work, a single ADC content (4 phr) was selected as the optimum determined from a previous study [38].

## 2. Materials and Methods

### 2.1. Materials

Poly (ethylene-*co*-octene) (PEO Engage-8150) containing 24 wt.% of octene with a melt index of 0.50 g/10 min (190 °C/2.16 kg, ASTM D1238), Mooney viscosity of 33 (ML 1 + 4, 121 °C, ASTM D1646), with 16% of total crystallinity and a density of 0.874 g/cm^3^ was supplied by the Dow Chemical Company (Midland, MI, USA). Azodicarbonamide (ADC) (AZ-760A) as a chemical foaming agent with a decomposition temperature range of 200–215 °C was purchased from Chempoint (Bellevue, WA, USA).

### 2.2. Preparation of POE-ADC Compounds

A laboratory twin-screw extruder was used for melt compounding of the POE with 4 phr of ADC. Compounding was performed using a co-rotating twin-screw extruder ZSE-27 (Leistritz, Allendale, NJ, USA) with an L/D ratio of 40 with D = 27 mm. The feeding rate was kept at 2 kg/h and the diameter of the circular die was 2.7 mm. The screw speed was fixed at 12 rpm with a flat temperature profile of 110 °C. The extrudate was cooled in an ice bath before being pelletized at ambient temperature.

### 2.3. Foam Preparation

A single-step foaming technique was used to produce the uniform and graded POE foams. First, 10 g of the compound was placed in a square mold with dimensions of 8 × 5 × 0.35 cm^3^. The temperature of the bottom (T_1_) and top (T_2_) plates of a hot press were preheated to specific temperatures as reported in Table 1 before the filled mold was placed between them for a total of 12 min under a pressure of 8.5 bar. For uniform samples (T205), T_1_ was set equal to T_2_, while for graded foams, different temperatures were imposed for both plates (T_1_ ≠ T_2_). In all cases, the highest temperature was set on the top plate. According to Table 1, the first series of graded samples (T210 to T225) had different Δ*T* and *T_avg_*, while the second series (dT10 to dT40) was prepared at fixed *T_avg_* and various Δ*T*. The side of the foam which was in contact with the highest temperature (T_2_) is referred to as the “Top” side, while the side in contact with the lowest temperature (T_1_) is called the “Bottom” side. In order to stabilize the cell structure, the mold was cooled to room temperature under pressure before opening for expansion. The unfoamed sample (neat matrix) was coded as PA0 (0 phr of ADC) and used as a reference to determine the effect of the cellular structure.

### 2.4. Characterization

The foam density was obtained by a gas pycnometer (UltraPyc 1200e, Quantachrome, Boyton Beach, FL, USA) using 3 replicates. Equation (1) was used to calculate the foam expansion ratio where the density of the unfoamed POE is 0.874 g/cm^3^ [39]:

(1) $$\mathrm{Expansion}\;\mathrm{ratio}\;\left(\%\right)=\left(1-\frac{Density\;of\;foamed\;sample}{Density\;of\;unfoamed\;sample}\right)\times 100$$

A scanning electron microscope (SEM) Inspect F50 (FEI, Hillsboro, OR, USA) was used to examine the foam morphology (cell structure) at 15 kV under different magnifications. The samples were first cryo-fractured (liquid nitrogen) and sputter coated with gold before imaging. The BELView software was used to quantify the foam morphology via several parameters, such as the number average cell size (*D_n_*), weight average cell size (*D_w_*), polydispersity index (*PDI*) and cell density (*ρ_cell_*) as [40,41,42,43]:

(2) $$D_{n}=\frac{\sum \left(n_{i}\cdot D_{i}\right)}{\sum n_{i}}$$

(3) $$D_{w}=\frac{\sum \left(n_{i}\cdot D_{i}^{2}\right)}{\sum (n_{i}\cdot D_{i})}$$

(4) $$PDI=\frac{D_{w}}{D_{n}}$$

(5) $$\rho_{cell}=\frac{\rho_{bulk}}{\rho_{foam}}\cdot {\left(\frac{\sum n_{i}}{A}\right)}^{\frac{3}{2}}$$

where *n_i_* represents the number of cells with a diameter *D_i_*, *A* is the foam surface analyzed, *ρ_bulk_* is the density of the unfoamed matrix and *ρ_foam_* is the foam density.

A PTC Instrument (ASTM D2240, model 307 L, Boston, MA, USA) was used to determine the foam hardness (Shore A). The average and standard deviation of 5 repetitions were used for each side of every sample. Tensile testing (ASTM D412) was conducted at room temperature (23 °C) on an Instron universal testing machine (USA) model 5565 with a 500 N load cell. The crosshead speed was 10 mm/min and the values (modulus, strength, etc.) were obtained by the average of a minimum of five samples. For the compression tests (ASTM D575), an RSA3 TA Instruments (USA) dynamic mechanical analyzer (DMA) was used with cylindrical samples (2.5 cm in diameter and 3.5 mm in thickness) and compressed at a rate of 0.01 mm/s. The elastic modulus was calculated in the linear zone of strain. All the properties reported represent an average of a minimum of three samples at room temperature. Finally, a home-made thermal conductivity analyzer based on ASTM E1225 was used to calculate the heat flux (*Q*) and determine the thermal conductivity (*k*) foams. The specimens were cut (5 × 5 cm^2^) and a digital caliper was used for measuring the thickness (*L* = 3.47–3.72 mm). Each sample was placed between two thin aluminum sheets and two plates with controlled temperatures of 33 °C (top plate) and 13 °C (bottom plate) giving an average room temperature of 23 °C and a temperature difference Δ*T* = 20 °C. Water-cooled Pelletier plates (Model K20, Haake, Vreden, Germany) kept the temperatures constant, while the equilibrium heat flux was measured via a PHFS-01 heat flux sensor (Flux Teq LLC, Blacksburg, VA, USA). The k values reported represent the average of three repetitions with their standard deviations calculated via the Fourier law as:

(6) $$k=\frac{QL}{\Delta T}$$

For mechanical compression and thermal conductivity, each sample was tested on both sides to determine any asymmetry due to the density gradation across thickness.

## 3. Results and Discussion

### 3.1. Morphological and Physical Characterization

#### 3.1.1. First Series

**Figure 1:**
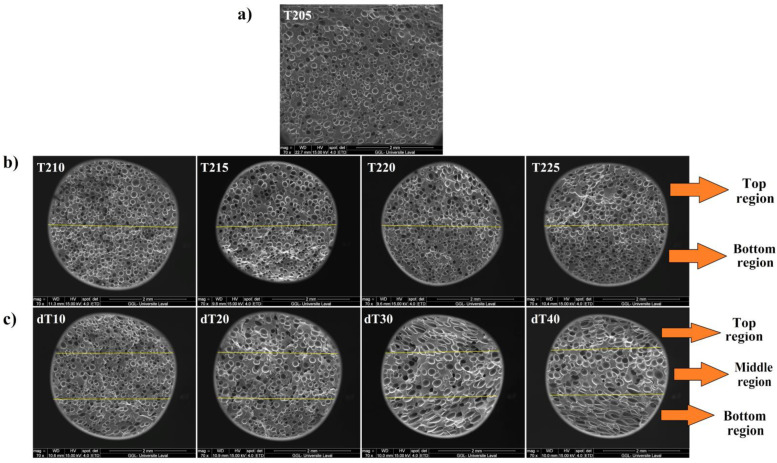
(**a**) SEM images of the uniform foam (T205) and graded POE foams: (**b**) first series and (**c**) second series (scale bar of 2 mm).

**Figure 2:**
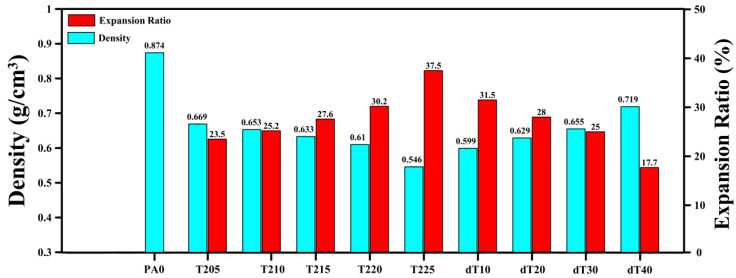
Density and expansion ratio of the unfoamed, uniform and graded POE foams.

Figure 1 presents typical SEM images of the uniform (Figure 1a) and graded POE foams with varying foaming temperatures (Figure 1b,c). The quantitative characterizations of these SEM images are reported in Table 2. Sample T205 has a homogeneous cell structure leading to a narrow PDI (1.022), low average cell size (105 µm) and high cell density (570 cells/mm^3^). The first series of samples (Figure 1b) led to the formation of two (top and bottom) regions with different cellular structure in terms of cell size and cell density across the foam thickness. The average cell size and cell density for the top and bottom regions in T210 are 117 and 107 µm, with 750 and 820 cells/mm^3^, respectively. Since the temperature difference in T210 is not very high (Δ*T* = 5 °C), there is limited difference between the cell size/density in both regions. However, the cellular structure difference between both regions is more significant at higher *T_avg_*. By increasing *T_avg_* from 207.5 °C (T210) to 215 °C (T225), the cell size in the top region increased from 117 to 154 µm (32%), while the values in the bottom region decreased from 107 to 104 µm (3%), respectively. Moreover, increasing *T_avg_* from 207.5 to 215 °C resulted in lower cell density in the top (56%) and bottom (3%) regions. The significant decrease in cell density in the top region is due to more gas volume being available with increasing temperature. This condition leads to higher internal cell pressure, enhancing the possibility of cell coalescence and collapse, leading to higher cell size and lower cell density. Furthermore, increasing *T_avg_* from 207.5 °C (T210) to 212.5 °C (T220) decreased the PDI in the top region from 1.018 to 1.009, while the values in the bottom region increased from 1.012 to 1.020, respectively. Higher *T_avg_* (from 212.5 to 215 °C) resulted in higher PDI in the top region (1.009 to 1.016) with lower values in the bottom region (1.020 to 1.010). To complete the morphological analysis, the foam density and foam expansion ratio are compared in Figure 2. The foam density decreased from 0.669 to 0.546 g/cm^3^ as *T_avg_* increased from 205 to 215 °C. The larger volume of gas produced at higher *T_avg_* is the main reason for this trend. For example, higher gas volume generated from the higher temperature between T205 and T225 led to higher expansion ratios of 24% and 38%, respectively.

#### 3.1.2. Second Series

The second series of samples led to the formation of three regions with different cell shape and cell characteristics: middle (circular cells) as well as top and bottom (elliptical/elongated shape) regions (Figure 1c). Because the top and bottom sections exhibited nearly identical cellular properties, they will be analyzed together. In other words, keeping *T_avg_* = 205 °C and increasing Δ*T* from 10 to 40 °C (dT10 to dT40) increased the cell size in the middle region from 98 to 176 µm, while the cell size in the top and bottom regions increased to 122 and 337 µm, respectively. As a result, the cell density in the middle region decreased by 47%, while it decreased by 52% in the top and bottom regions (Table 2). As mentioned for the first series, larger cell size and lower cell density are related to higher possibilities of cell coalescence and collapse due to higher generation of gas at elevated temperatures. Furthermore, the cell size difference between the middle region and both top/bottom regions increased with increasing Δ*T*. For instance, the cell size difference in dT10 between the regions is only 24 µm, while it increases to 161 µm for dT40. In our case, the highest temperature was applied to the top plate, hence, increasing the temperature leading to higher/faster ADC decomposition locally, generating a larger volume of gas. Thus, more gas molecules are available nucleating a higher number of cells with higher internal cell pressure. This can eventually increase the local probability of cell coalescence and collapse [44]. Additionally, the dissolved gas molecules have a stronger plasticizing effect, decreasing the elasticity and viscosity of the matrix, making it easier for the rupture of cell walls during the foaming process, especially as the temperature is high [45]. Further, increasing Δ*T* from 10 to 20 °C (dT10 to dT20) led to higher PDI in the middle region (from 1.018 to 1.022), as well as in the top and bottom regions (from 1.030 to 1.035), while the values from Δ*T* = 20 to 40 °C (dT10 to dT20) decreased in the middle region (from 1.022 to 1.016), as well as in the top and bottom regions (from 1.035 to 1.015), respectively. According to Figure 2, the foam density increased from 0.599 to 0.719 g/cm^3^ as Δ*T* increased from 10 to 40 °C, while the expansion ratio decreased from 32 to 18% from dT10 to dT40. This trend is related to the higher probability of cell rupture/coalescence at higher Δ*T* [46,47].

### 3.2. Mechanical Properties

#### 3.2.1. First Series

The tensile results for the unfoamed, uniform and graded POE foams are shown in Table 3. Two main factors are known to control the foams’ behavior: foam density (ρ_foam_) and morphology (D_n_, PDI and ρ_cell_). According to the results obtained, it is obvious that higher *T_avg_* results in lower mechanical properties (tensile modulus, strength, elongation at break and hardness) due to lower sample density. The uniform foam (T205) has lower modulus (0.7 MPa), strength (2.55 MPa) and elongation at break (939%) compared to the unfoamed matrix. Increasing *T_avg_* (205–210 °C) reduced the modulus, strength and elongation at break even more by 17%, 8% and 10%, respectively. This is mostly associated with the presence of a higher volume of gas (bubbles/voids) inside the matrix produced at higher processing temperature (*T_avg_*). The tensile properties of the other graded foams have a similar trend as they decrease with increasing *T_avg_* (Table 3). For instance, increasing *T_avg_* from 210 to 215 °C decreased the tensile modulus (0.58 to 0.44 MPa) and strength (2.35 to 2.18 MPa), indicating that only a 5 °C difference can have a substantial effect on the mechanical properties.

**Figure 3:**
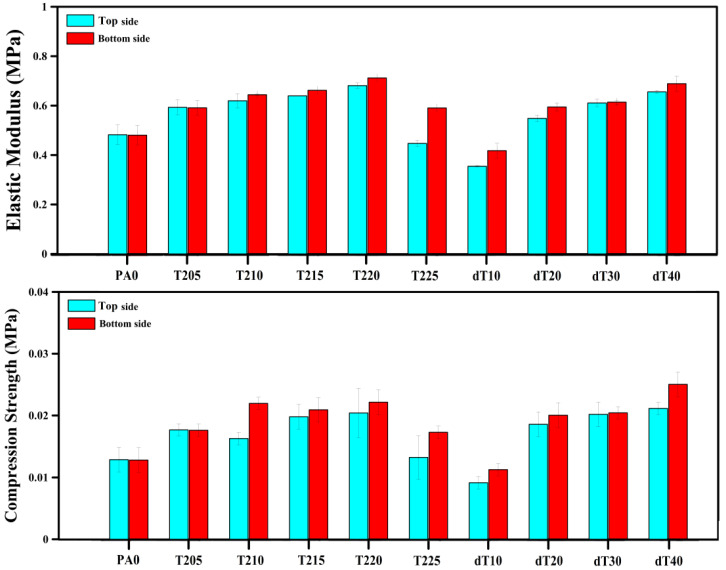
Elastic modulus and compression strength (at 7% compression) of the unfoamed, uniform and graded POE foams.

**Figure 4:**
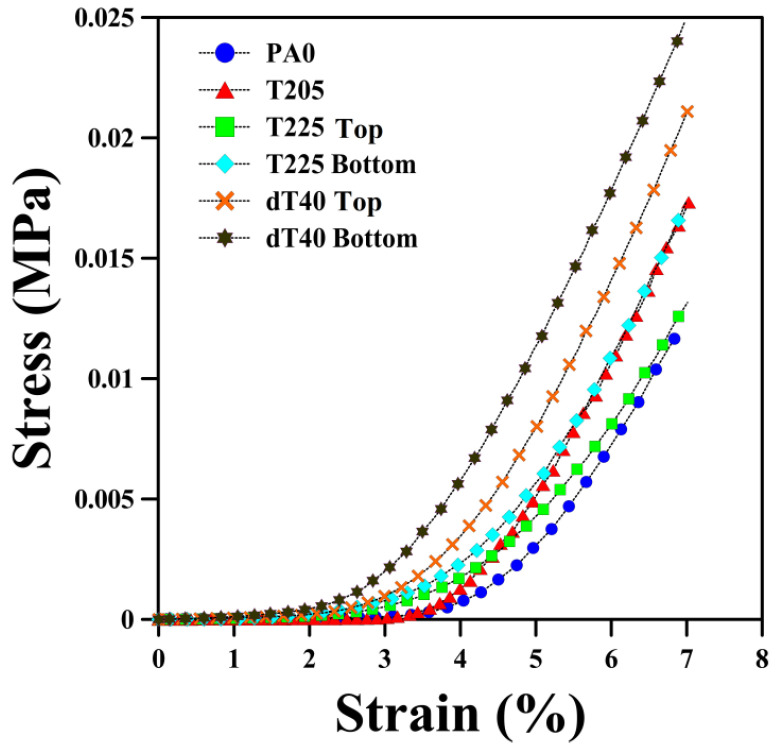
Compressive stress–strain curves of unfoamed (PA0), uniform (T205) and graded POE foams (top and bottom sides of T225 and dT40).

The compression test was carried out on both sides of each sample and the values with typical stress–strain curves (PA0, T205, T225 and dT40) are reported in Figure 3 and Figure 4, respectively. The results show that the compression elastic modulus and compressive strength (at 7% compression) increased by 15% and 16% (top side), but 20% and 26% on the other side (bottom side), respectively, at lower *T_avg_* (205–212.5 °C). This is due to the higher *T_avg_* leading to the production of more gas molecules inside the matrix. As each cell acts as an inflated balloon, the presence of more gas volume/pressure inside the cells improves their resistance against compressive forces [48]. In addition, foaming the POE led to higher compression properties. For instance, the elastic modulus increased from 0.482 to 0.593 MPa between PA0 and T205, respectively. However, both elastic modulus (34% top and 17% bottom side) and compressive strength (35% top and 22% bottom side) decreased at higher *T_avg_* (212.5–215 °C). This behavior is directly related to morphological changes in *D_n_* and *ρ_cell_* (Table 2), as previously discussed [49]. As reported in Figure 3 and Figure 4, the bottom side of graded foams (having lower cell size and higher cell density) is more resistant to compression force. For example, the bottom side of T225 has higher elastic modulus (33%) and compression strength (31%) compared to its top side.

**Figure 5:**
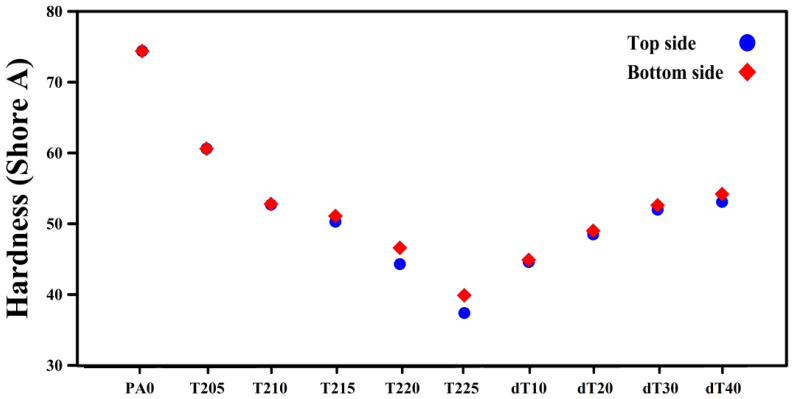
Hardness of unfoamed, uniform and graded POE foams (top and bottom sides).

Finally, the foams’ hardness (Shore A) decreased by 36% (60.6 to 38.7) by increasing *T_avg_* from 205 to 215 °C (Table 3 and Figure 5). The most significant factor contributing to the loss of hardness is the lower foam rigidity and resistance to needle penetration associated with an increase in expansion ratio (lower density) at higher *T_avg_* as less material is available to sustain the applied stresses. Analyzing the hardness for both sides of each foam also showed some differences. This difference was negligible for the foams prepared at lower *T_avg_* (205–207.5 °C), but the difference increased from 2% to 7% for higher *T_avg_* (210–215 °C), respectively. Nevertheless, the hardness difference could also be associated with differences in the crosslink density of the matrix. This was not determined here but will be investigated in future studies.

#### 3.2.2. Second Series

The mechanical properties of the second series showed a different trend compared to the first series (Table 3 and Figure 3, Figure 4 and Figure 5) as all the mechanical properties (tensile, compression and hardness) were improved with increasing Δ*T*. For example, increasing Δ*T* from 10 to 40 °C produced higher modulus (14%), strength (26%) and elongation at break (10%). The reason for this trend was discussed above and is related to higher density having more material available to withstand the applied stress.

Similar to the first series’ trend, the elastic modulus and compressive strength of the second series increased by 85% and 131% (top side), as well as 65% and 121% (bottom side) from dT10 to dT40, respectively (Figure 3 and Figure 4). According to SEM images (Figure 1c), increasing Δ*T* led to the formation of three regions (top, middle and bottom), where the presence of these three regions plays a significant role in resisting against compressive loads. The presence of elliptical cells with large cell sizes (high volume of gas), at the top and bottom regions, prevents the sample from being compressed. Furthermore, the middle region, with homogenous and smaller cells, helps the sample to improve its resistance against compressive loads [50]. Finally, the hardness of the foams increased by 20% by increasing the Δ*T* from 10 to 40 °C (Figure 5). The highest hardness difference between both sides of a sample was obtained for dT40. Again, here, the results clearly showed that a graded morphology led to asymmetric mechanical properties. In our case, the maximum difference was 45% for the compression strength of dT20.

### 3.3. Thermal Conductivity

#### 3.3.1. First Series

The thermal conductivity of uniform and graded POE foams was carried out on both sides of each foam and Table 4 reports on the values obtained. As expected, the *k* value of the uniform foam was the same (0.165 W/m.K) for both sides. In the first series of sample, increasing *T_avg_* from 207.5 to 215 °C decreased the *k* value by 22% (from 0.161 to 0.125 W/m.K). This is mainly related to higher expansion ratio (lower density) as more gas (cells) inside the foam provides better thermal insulation for the foams [51,52]. Furthermore, Table 4 shows that the top side of graded foams has higher *k* values than the bottom side. This indicates that the bottom side (lower cell size and higher cell density) generates lower thermal conductivity. For example, the bottom side of T220 has 4% higher thermal insulation compared to its top side. It can be concluded that the presence of a higher population of cells (cell density) with lower cell size leads to lower thermal conductivity.

#### 3.3.2. Second Series

The thermal conductivity of the second series showed a different trend compared to the first series. By increasing Δ*T* from 10 to 40 °C, the *k* value increased by 7% (0.169 to 0.180 W/m.K). The main reason for this behavior is attributed to lower expansion ratio (lower amount of gas inside the foam). The *k* values obtained from both sides did not show significant differences for these samples. This can be attributed to the similar morphology of the top and bottom regions (elliptical). A comparison between the thermal insulation performance of the first and second series shows that the cell size and cell density are both critical parameters for heat transfer. For instance, comparing the top and bottom regions of dT40 and T225 shows that the former has larger cell sizes (54% for the top and 69% for the bottom regions) and lower cell density (61% for the top and 290% for the bottom region). This is the origin of the higher thermal insulation of T225 (44%) compared to dT40.

## 4. Conclusions

In this work, uniform and density-graded foams were produced using a single-step compression molding process. POE was used as an elastomeric matrix with single blowing agent (ADC) content (4 phr). To control the final morphology of the foams, two series of graded foams with different *T_avg_* and Δ*T* were produced, leading to the formation of different (two or three) regions, respectively, with different cellular morphology (cell size, density and geometry). The results were compared with uniform foam and the neat (unfoamed) polymer matrix.

The cellular structure analysis of the first series indicated that increasing *T_avg_* from 207.5 °C to 215 °C resulted in higher cell size in the top region (32%) and lower cell size in the bottom region (3%) combined with lower cell density in the top (56%) and bottom (3%) regions. On the other hand, increasing Δ*T* from 10 to 40 °C led to larger cell sizes (80%) in the middle region, as well as larger cell sizes in the top and bottom regions (175%).

The tensile properties, such as modulus, strength and elongation at break, of the first series decreased by 33%, 13% and 15%, respectively, with increasing *T_avg_*, while they increased for the second series by 14%, 26% and 10%, respectively, with increasing Δ*T*. For compression, the tests were carried out on both sides of the foams to detect any asymmetry related to the density gradation. Surprisingly, the graded structure of the second series produced a significant improvement in the elastic modulus (85%) and compressive strength (131%) on the top side compared to only 65% and 121% for the bottom side from dT10 to dT40, respectively.

Finally, the thermal conductivity of the first series was decreased by 22%, while it increased by 7% for the second series. Moreover, the thermal insulation analysis of both sides revealed that the side having smaller cell size and higher cell density had better overall thermal insulation performance. This is why the first series of graded foams showed lower thermal conductivity than the second series. The thermal conductivity behavior of the graded POE foams revealed that the amount of thermal damping of the foams depends on two main factors: expansion ratio/foam density (the amount of gas inside the matrix) and the geometry/cellular structure. Therefore, by carefully engineering and tailoring the cellular morphology of the foam, their thermal insulation behavior can be optimized for a fixed amount of materials and be used as high-efficiency thermal insulators in buildings. In addition, since the temperature difference between the inside and outside of buildings in cold and tropical regions can vary over a wide range of temperature differences, we plan to measure the thermal conductivity of graded POE foams at different average temperatures (above and below room temperature). Finally, the real 3D structure of the foams can be simulated via 2D SEM images, which will be applied in finite element techniques (FEM) to simulate the heat transfer behavior of the graded POE foams.

## Figures and Tables

**Table 1:** The temperatures and codes of the samples prepared.

Sample Code	T_1_ (°C)	T_2_ (°C)	ΔT = T_2_ − T_1_ (°C)	*T_avg_* (°C)
T205	205	205	0	205
T210	205	210	5	207.5
T215	205	215	10	210
T220	205	220	15	212.5
T225	205	225	20	215
dT10	200	210	10	205
dT20	195	215	20	205
dT30	190	220	30	205
dT40	185	225	40	205

**Table 2:** Morphological characteristics of the uniform and graded POE foams.

**Sample**	**D_n_** **(µm)**		**D_w_** **(µm)**		**PDI** **(-)**		**Cell Density** **(cells/mm^3^)**	
	**Top**	**Bottom**	**Top**	**Bottom**	**Top**	**Bottom**	**Top**	**Bottom**
T205	105	105	107	107	1.022	1.022	570	570
T210	117	107	119	108	1.018	1.012	750	820
T215	137	102	138	104	1.006	1.013	440	550
T220	149	102	151	104	1.009	1.020	260	640
T225	154	104	157	105	1.016	1.010	330	800
**Sample**	**D_n_** **(µm)**		**D_w_** **(µm)**		**PDI** **(-)**		**Cell density** **(cells/mm^3^)**	
	**Middle**	**Top and Bottom**	**Middle**	**Top and Bottom**	**Middle**	**Top and Bottom**	**Middle**	**Top and Bottom**
dT10	98.0	122	99.7	126	1.018	1.030	680	430
dT20	128	139	131	144	1.022	1.035	320	290
dT30	166	286	169	292	1.017	1.022	380	204
dT40	176	337	179	342	1.016	1.015	360	205

**Table 3:** Tensile and hardness results for unfoamed, uniform and graded POE foams.

Sample	Tensile Properties			Hardness		
	Modulus @ 100% (MPa)	Strength @ Break (MPa)	Elongation @ Break (%)	(Shore A)		
				Top	Bottom	Average
PA0	1.11 ± 0.01	6.48 ± 0.91	1290 ± 98	74.4 ± 0.3	74.4 ± 0.3	74.4
T205	0.70 ± 0.03	2.55 ± 0.29	939 ± 43	60.6 ± 0.3	60.6 ± 0.3	60.6
T210	0.66 ± 0.01	2.49 ± 0.10	881 ± 10	52.7 ± 0.5	52.8 ± 0.4	52.8
T215	0.58 ± 0.02	2.35 ± 0.10	846 ± 10	50.3 ± 0.5	51.1 ± 0.6	50.7
T220	0.55 ± 0.02	2.20 ± 0.01	782 ± 16	44.3 ± 0.5	46.6 ± 0.4	45.5
T225	0.44 ± 0.01	2.18 ± 0.10	746 ± 28	37.4 ± 0.5	39.9 ± 0.4	38.7
dT10	0.57 ± 0.03	2.21 ± 0.10	906 ± 7	44.6 ± 0.5	44.9 ± 0.4	44.8
dT20	0.61 ± 0.03	2.25 ± 0.07	941 ± 11	48.5 ± 0.3	49.0 ± 0.4	48.8
dT30	0.63 ± 0.05	2.66 ± 0.08	972 ± 8	52.0 ± 0.6	52.6 ± 0.5	52.3
dT40	0.65 ± 0.09	2.78 ± 0.04	992 ± 9	53.1 ± 0.7	54.2 ± 0.7	53.7

**Table 4:** Thermal conductivity of the unfoamed, uniform and graded POE foams.

Sample	k (W/m.K)		
	Top	Bottom	Average
PA0	0.193 ± 0.001	0.193 ± 0.001	0.193
T205	0.165 ± 0.002	0.165 ± 0.002	0.165
T210	0.162 ± 0.004	0.160 ± 0.006	0.161
T215	0.158 ± 0.001	0.154 ± 0.003	0.156
T220	0.143 ± 0.002	0.137 ± 0.002	0.140
T225	0.128 ± 0.001	0.121 ± 0.002	0.125
dT10	0.169 ± 0.005	0.168 ± 0.001	0.169
dT20	0.172 ± 0.002	0.170 ± 0.003	0.171
dT30	0.176 ± 0.001	0.174 ± 0.002	0.175
dT40	0.181 ± 0.001	0.178 ± 0.002	0.180

## Data Availability

The data presented in this study are available on request from the corresponding author.
